# Parental Stress and Children’s Self-Regulation Problems in Families with Children with Autism Spectrum Disorder (ASD)

**DOI:** 10.3390/jintelligence10010004

**Published:** 2022-01-17

**Authors:** Maria Efstratopoulou, Maria Sofologi, Sofia Giannoglou, Eleni Bonti

**Affiliations:** 1 Department of Special Education (CEDU), United Arab Emirates University (UAEU), Al Ain P.O. Box 15551, United Arab Emirates; maria.efstratopoulou@uaeu.ac.ae; 2 Laboratory of Psychology, Department of Early Childhood Education, School of Education, University of Ioannina, 45100 Ioannina, Greece; m.sofologi@uoi.gr; 3 Institute of Humanities and Social Sciences, University Research Centre of Ioannina (URCI), 45110 Ioannina, Greece; 4 First Psychiatric Clinic, School of Medicine, Faculty of Health Sciences, Aristotle University of Thessaloni-ki, “Papageorgiou” General Hospital, Ring Road Thessaloniki, N. Efkarpia, 54603 Thessaloniki, Greece; sophiegianno@gmail.com; 5 Department of Education, School of Education, University of Nicosia, Nicosia 2417, Cyprus

**Keywords:** parental stress, self-regulation, social support, coping strategies, ASD, behavioral difficulties, non-verbal children

## Abstract

*Background*: Increased parental stress is strongly related to the severity of autism spectrum disorder (ASD) symptomatology. Parents’ coping strategies and social support issues add to the complexity of this relationship. *Aim*: The present study investigated the relationship between self-regulation skills and parenting stress in parents of nonverbal children with ASD. *Methods and procedure*: The Parenting Stress Index–Short Form (PSI-SF) was administered to 75 families, and self-regulation scores on a Motor Behavior Checklist for children (MBC) were recorded by students’ class teachers (level of functioning-behavioral problems). In addition, interviews were conducted with a focus group of six parents (four mothers and two fathers) to explore the underline factors of parental stressin-depth. *Results*: Correlation analyses revealed that parenting stress was positively correlated with elevated scores on MBC children’s self-regulation subscale. On the other hand, parenting stress was negatively correlated with the level of social functional support reported. Qualitative data were analyzed using transcripts, revealing additional stressors for families and parents, and resulting in recommendations to overcome these factors. *Conclusions and implications*: Aiming at developing strategies to improve self-regulation skills in nonverbal children with ASD may be particularly important in reducing parental stress for families having nonverbal children with autism and other developmental disabilities. Parents’ stressors and suggestions during interviews are also discussed.

## 1. Introduction

Family schema is a dynamic living cell. Parents as caretakers play a vital role inmentoring and guiding their children’s learning journey from early childhood into adulthood. Parental stress is an anxiety closely aligned with the significant role of being a parent. However, when it comes to parents of children with autism spectrum disorder (ASD), this role often starts as soon as their child is diagnosed (Foster et al. 2012). More specifically, parents of children with ASD very often face a plethora of difficulties regarding the educational opportunities focused on developing their skills and achieving a high quality of life (Meadan et al. 2010; Tincani et al. 2014). In this vein, parents are faced with a range of emotional pressures as they attempt to learn about ASD and what this means for their child. Compared with parents of children with intellectual or other developmental disabilities, studies reveal that parents of children with ASD experience more psychological distress, including depression, anxiety, and components of stress, such as decreased family cohesion and increased somatic symptomatology (Martins et al. 2015).

### 1.1. Autism Diagnosis and Parenting Stress

An autism diagnosis not only changes the life of the child diagnosed but also the quality of emotional harmony of all family members. Parents describe experiencing initial feelings of surprise, sadness, shock, and rejection following their child’s diagnosis (Martins et al. 2015). It is an axiom to realize that the family, as a holistic schema, is facing and must cope with the challenges of communicating with and assisting the social interactions of their child with ASD. At this point, it is essential to underline that parents have to manage high levels of anxiety owing to perplexing therapeutical programs, such as home-implemented treatments and juggling job responsibilities, as well as family commitments (Hayes and Watson 2013). Thus, the diagnosis of a child with autism spectrum disorder (ASD) can be a time of tremendous uncertainty, agony, and a depressive journey for parents and families. Parents must handle a maze of information and bureaucratic processes as they attempt to find the appropriate therapeutic intervention program to strongly support their child (Tomeny 2017).

There is a great variety of therapeutic approaches, which focus on accommodating or remediating different challenges related to autism. The therapeutic approach is effective only when it is individualized depending on the chronological age and developmental level; therapies focus on all difficulties, the interactions between them, and the promotion of growth and adaptation in the long run (Poppi et al. 2019). In Greece, around the time that parents start to come to terms with their child’s condition, they have to decide what kind of treatment plan they are going to follow. Children with autism are considered to be very different from each other, and the clinical presentation of their symptoms varies along with the outcomes following an intervention (Ben-Itzchak et al. 2014). There are many different treatments provided to children with autism, such as the treatment and education of autistic and related communication-handicapped children (TEACCH), cognitive behavioral therapy, applied behavior Analysis (ABA), learning experiences: an alternative program for preschoolers and parents (LEAP),picture exchange communication systems (PECS),speech and language therapy, occupational therapy, etc.(Poppi et al. 2019; Ben-Itzchak et al. 2014; CDC 2021).

Several significant factors are closely aligned with anxiety or agony in parents of children with ASD (e.g., child characteristics, lack of emotional and social support) (Lee et al. 2009). Although research reveals an impact on family members of individuals with ASD, Hastings et al. (2005a) emphasize that not all family members experience similar effects as a result of having an individual with ASD in the family. For example, Hastings (2003) found that mothers of children with ASD reported more anxiety and negative outcomes than fathers in the same family. In addition, researchers have found positive outcomes (e.g., limited conflicts within the relationship, high self-esteem and self-concept) for some typically developing siblings of individuals with ASD (Sharabi and Marom-Golan 2018), whereas some parents described the experience of having a child with ASD as being positive (Hastings et al. 2005a).

Under the scope of evaluating parental levels of anxiety, we must underline the fact that, although the research community has focused on subgroups referred to as “high-functioning autism” (Baio et al. 2018), the influence of the child’s IQ (intellectual quotient) level on family functioning has hardly been studied. Around 70% of parents of children with Asperger syndrome score at or above the 90th percentile of the normal parental stress scores (Matson and Kozlowski 2011). It appears that a high level of cognitive development in children with ASD does not, in itself, influence and reduce the stress produced by raising a child with ASD. There is a research consensus emphasizing the fact that parents of children with high-functioning autism report significantly higher levels of long-term agony and lower levels of adaptive coping strategies and resources when compared with parents of children with typical development (Hayes and Watson 2013). Furthermore, concerning the lifelong nature of ASD, these challenges are often longstanding and extend into the sons’ and daughters’ adolescense and adulthood. Moreover, parents confirm that much of their stress and emotional exhaustion is caused by the continued necessity of having to fight for services, cope with complicated policies or negative societal attitudes, and constantly having to communicate and build relationships with education and health professionals (Yorke et al. 2018).

Undoubtedly, an increased tendency to experience negative emotions and a decreased ability to regulate emotional responses appear common to many childhood psychiatric and developmental disorders—although, not surprisingly, the links between other temperamental traits and psychopathology vary by disorder (Nakagawa et al. 2016). The research community has consistently identified neurodevelopmental disorders as being linked to specific temperament configurations (Johnson et al. 2014). Several researchers also include activity level, attentional control, and impulsivity as temperament dimensions. According to the above conceptualizations, it should come as little surprise that the association between emotional problems and lack of self-regulatory mechanisms in children with ASD and parental stress is strong enough to warrant speculation that the disorder is perhaps better understood dimensionally.

In conclusion, the perplexing phenomenon of parenting stress in families of children with ASD requires a holistic approach to thoroughly evaluate the possible influence of multiple dimensions simultaneously. The age range and level of cognitive development of the individuals with ASD in the samples have been quite heterogeneous (Hastings et al. 2005b; Manning et al. 2011; Pozo and Sarriá 2014a, 2014b; Zaidman-Zait et al. 2014, 2018; Giovagnoli et al. 2015). However, so far, sporadic studies have investigated the predictors of parental stress in school children with ASD without intellectual disability (Bundy and Kunce 2009; Lee et al. 2009; Mori et al. 2009; Craig et al. 2016).

### 1.2. Hypothesis of the Present Study

Under the aegis of the above theoretical and research findings, the current study attempts to evaluate two different objectives. The first purpose of the study was to assess the complex relationship between parental stress and the severity of the ASD symptoms, behavioral difficulties, coping strategies, and social support. The second objective of the study was to evaluate the influence of behavioral difficulties within the sphere of temperament, coping strategies, and social support between ASD symptoms and parenting stress. We hypothesized that parenting stress would be positively correlated with ASD symptom severity, the temperament of the children, and coping strategies related to distraction and disengagement (Hypothesis 1). In addition, parenting stress would be negatively correlated with engagement and cognitive reframing coping strategies and social confidence and affective functional support (Hypothesis 2). Additionally, a harmonic balance among individuals and their environment is produced through a two-way interaction between inherent and temperamental traits and external experiences and circumstances, as well (Rothbart 2007).

## 2. Method

### 2.1. Participants

For the present study, 75 parents with at least one child with an autism diagnosis participated in the survey (M = 36.2, SD = 8.9). All the families lived in Greece and were recruited through special public schools and parental support groups. A member of the research team contacted the parents to explain the objectives of the study and request their collaboration. The children had received a clinical diagnosis of an autism spectrum condition in the psychiatry and child neurology services of public hospitals and medical centers in Greece, at ages ranging between 2 years and 11 months and 6 years (mean age of diagnosis = 4.69; SD = 1.67). The diagnosis was generally made using a multi-team approach, based on the Diagnostic and Statistical Manual of Mental Disorders (DSM-5; APA 2013) and the Autistic Diagnostic Interview—Revised (ADI-R; Rutter et al. 2006).

Finally, 18 class teachers with a mean age (years) M = 32.15 (SD = 3.62) working with the children in public primary special schools completed the Motor Behavior Checklist to rate the self-regulation skills of their students.

Inclusion criteria: (1) families with at least one child with an autism diagnosis; (2) families that live in Greece; (3) children with an autism diagnosis, either secondary or idiopathic autism; (4) children diagnosed from public hospitals and medical centers; (5) children in primary special public schools; (6) children older than 6 yearsand up 12 years (primary school); (7) parents that are participating in parental support groups.

Exclusion criteria: (1) children diagnosed with ASD from private practices and not from public hospitals and medical centers; (2) children not in primary special public schools; (3) children aged younger than 6 years or older than 12 years; (4) families not in parental support groups; (5) families not living in Greece.

### 2.2. Assessment Instruments

#### 2.2.1. Parenting Stress Index–Short Form

Parenting Stress Index–Short Form (PSI-SF; Abidin 1995; Adapted to Greek) was used in this research. This scale was a self-report measure filled out by the parents. It contained 36 items distributed in three subscales of 12 items each, rated on a five-point Likert-type response scale. The first scale, parental distress, evaluated the distress experienced by parents due to personal factors, such as depression or conflict with a partner, or life restrictions due to the demands of childrearing in their role as parents (i.e., “Since having my child, I feel that I am almost never able to do things I like to do”). The second scale, parent–child dysfunctional interaction, provided information on the parents’ feelings about the interactions with their child and the degree of frustration of the expectations and trust they have placed in their child (i.e., “Most times, I feel that my child does not like me and does not want to be close to me”). The third scale, difficult child, was designed to measure the parents’ perceptions of their child’s self-regulatory abilities (i.e., “My child seems to cry or fuss more often than most children”). The scale also provided a measure of total stress by adding up the scores on the 36 items, with a total score above 90 being clinically significant. The Cronbach’s alpha internal consistency coefficients in our sample were parental distress (a = 0.91), dysfunctional parent–child interaction (a = 0.82), and difficult child (a = 0.90); this was similar to those obtained in other studies carried out in Spain (Diaz-Herrero et al. 2011). It is the most widely used instrument to evaluate stress in studies on ASD; in fact, it was utilized in 75% of the studies included in a recent systematic review (Barroso et al. 2018).

#### 2.2.2. Motor Behavior Checklist for Children

In the present study, the self-regulation skills of ASD children were assessed using the Motor Behavior Checklist’ (MBC) for children (Efstratopoulou et al. 2012b). The MBC checklist is a screening instrument designed to measure externalizing and internalizing behavioral symptoms of primary school-aged children. The instrument has been used in studies in Greece and has internal consistency (0.82 to 0.95), reproducibility (0.85 to 0.90), and interrater agreement (0.75 to 0.91) that have been checked in previous studies. More specifically, the MBC includes seven scales, which assess particular emotional and/or behavioral problems (i.e., rule breaking—7 items; hyperactivity/impulsivity—14 items; lack of attention—10 items; low energy—4 items; stereotyped behaviors—2 items; lack of social interaction—10 items; and lack of self-regulation—12 items). Many of these categories of behavioral problems can be observed in the form of both deficits and excesses in attention deficit hyperactivity disorder (ADHD) and autistic spectrum disorders (ASD) (Efstratopoulou et al. 2012a). The MBC should be completed by observing the child in a free-play situation or during physical education classes. The score is obtained through a 5-point Likert scale ranging from “never” (0) to “almost always” (4). Efstratopoulou et al. (2012a), evaluated the psychometric properties of the MBC: the coefficients of internal consistency (α) ranged from 0.82 to 0.95, reproducibility according to intraclass correlation coefficients (ICC) ranged from 0.85 to 0.90, and concordance (also ICC) ranged from 0.75 to 0.91. These data suggest that the MBC for children is a homogeneous instrument in terms of content, with high stability and correlation (Efstratopoulou et al. 2012a). In addition, results from several studies on the psychometric properties of the checklist indicated that the MBC is a useful tool to discriminate between the core symptoms of ADHD, conduct disorder, and ASD (Efstratopoulou et al. 2012b). For the purposes of this study, mean scores on self-regulation items were calculated for all ASD children by their class teachers.

#### 2.2.3. Child Autism Symptoms ASD Clinical Criteria from the DSM-5

The severity of the ASD symptoms was assessed through an interview between the parents and a clinical psychologist, focused on the seven diagnostic criteria for the disorder in accordance to DSM-5 (APA 2013). The first three were to evaluate socio-communicative impairments and the other four to rate repetitive behaviors and restricted interests. Through the interviews, the parents evaluated the severity of each criterion, using a 4-point Likert scale ranging from 0 to 3, where 0 represents “almost never”, 1 “sometimes”, 2 “often”, and 3 “many times”. Therefore, a higher score on the DSM-5 indicates greater severity of the ASD symptoms.

### 2.3. Procedure

All parents and teachers participated in the study voluntarily. Each parent received a file which contained: (a) the information letter concerning the objectives of the research, as well as the contact details of the research supervisor; (b) the form for completion of the demographic data; and (c) the Greek version of the Parenting Stress Index–Short Form questionnaire (PSI-SF; Abidin 1995). All participants were examined individually by the researcher who explained the procedure and conducted a small interview with them about the confidant and affective support they have in their everyday lives. Each parent had the opportunity to choose the place, as well as the time, to complete the questionnaires while at the same time, there was the possibility for clarifications, where this was necessary. No time limit was assigned for the completion of the questionnaire. Through the information letter, parents were encouraged to respond honestly, to ensure the reliability of the results. The participants gave written informed consent at the time of their visit, agreeing that their participation was voluntary and that they could withdraw at any time, without giving a reason and without cost. Due to the specific type of current research, demographic data such as age, gender, or occupation were selected. Since these are considered personal data, the European Union law that has existed since 28 May 2018 was applied.

According to the law, the use of sensitive personal data is allowed only due to research reasons. Therefore, the participants were informed accordingly, and they agreed that their personal data could be deleted from the web-database after a written request. This study obtained ethical approval and the participants’ parents, after being informed about the objectives, signed the consent forms to participate in the study. They were fully aware that they could leave if they so desired. Next, an individual brief interview was administered by an accredited psychologist to confirm the diagnosis, record the social support for the family and complete the symptom severity list with the parent, in order to extract the necessary data on their children to carry out this study. Finally, all teachers completed the Greek Version of the Motor Behavior Checklist for Children (Efstratopoulou et al. 2012b).

### 2.4. Data Analyses

The statistical analyses for the current study were performed using the IBM SPSS Statistics software, Version 24.0 (Statistical Package for Social Science). To examine the relationship between self-regulation problems in ASD children and parental stress, Pearson correlations were calculated. The bivariate Pearson correlation produces a sample correlation coefficient, r, which measures the strength and direction of linear relationships between pairs of continuous variables. By extension, the Pearson correlation evaluates whether there is statistical evidence for a linear relationship among the same pairs of variables in the population, represented by a population correlation coefficient, ρ (“rho”). The Pearson correlation is a parametric measure.

In the second step of statistical analysis, in order to evaluate possible gender differences on a total parental stress scores, a one-way ANOVA was applied between mothers and fathers.

## 3. Results

With regard to the ASD children, most of them were boys (63%), and their ages ranged from 7 to 11, with a mean age of 8.59 (SD = 1.38). The children attended classes in special primary public schools, and they were receiving extra educational support of varying degrees (20 out of the 45 children were enrolled in communication and language classrooms). 

In terms of parents and family characteristics, the parents’ mean age (years) was 40.17 (SD = 4.82). With regard to their education level, 40 of the participants (53.3%) had obtained a university degree, 30 participants (40%) had obtained a high school diploma, and five participants (6.7%) had studies corresponding to primary education. The majority of the parents were employed (67%), whereas nine reported that they were unemployed at the moment, or they had never worked outside the house/family. With regard to their marital status, most of them were married—60 participants (80%)—and the rest (15) were separated/divorced (20%). 

All demographic characteristics of parents are presented in Table 1.

To test “Hypothesis 1” and “Hypothesis 2”, Pearson correlation coefficients among research variables were estimated. The Pearson product–moment correlation analyses revealed the existence of significant associations between the ASD symptoms from the DSM-5, and the parenting stress total index (*p* < 0.001), Strengths and Difficulties Questionnaire (SDQ) (*p* < 0.001), engagement (*p* < 0.001), confidant support (*p* < 0.001), and affective support (*p* < 0.05). In addition, as shown in Table 2, the analyses revealed significant correlations between the parenting stress total index and the SDQ (*p* < 0.001), engagement (*p* = 0.04), and confidant support (*p* = 0.05). Self-regulation scores and scores on the separate subscales of the Parental Stress Index scale were calculated. Intercorrelations between variables are presented in Table 2.

There were significant correlations between scores on self-regulation problems reported by the teachers and the total parental stress scores (r = 0.713) and the mean scores on the subscale difficult child (r = 0.761) reported by the parent.These results mean that the severity of self-regulation problems experienced by children with ASD influenced and increased parental stress.

In the second step of statistical analysis, research findings revealed that there were no statistical differences in total scores on stress between mothers and fathers for our sample F (2,72) = 10.951, *p* < 0.001. However, the Pearson correlation revealed that the educational level of parents was positively correlated with the level of parent–child dysfunction (r = 0.761) and the total stress exhibited by the parent (r = 0.654).

### 3.1. Interviews with Parents

A focus group of six parents (four mothers and two fathers) participated in structured interviews. A simple random sampling was used for the selection of the group, and every individual had an equal chance of being selected. A transcendental psychological phenomenology qualitative approach proposed by Moustakas (1994), in which each experience stands in its unique features and the phenomenon is introduced in a fresh complete description of thoughts, perceptions, and feelings, was used. The approach derives “*a textural description of the meanings and essences of the phenomenon, the constituents that comprise the experience in consciousness, from the vantage point of an open self*” (p. 35). This implies the subjectivity of the researcher and his/her role in presenting the importance of the phenomenon (Moustakas 1994). The interview recordings were transcribed and analyzed using Moustakas’s (1994) phenomenological approach and Creswell and Poth’s (2018) approach. First, the interview data were prepared for analysis by transcribing the audio and videotaped interviews. Significant statements or quotes from the transcripts that were essential for understanding the phenomenon were highlighted and coded (horizontalization) based on the main themes exploring the participants’ experience (textural description) and how they experienced this phenomenon (structural description providing the themes with the retrieved quotes from the transcripts). Finally, the transcripts were sent to an external reviewer, who examined the themes and reflected on the validity and suitability of the themes to the purpose of the study and its questions.

#### 3.1.1. Findings from the Interviews

The official ASD diagnosis evoked negative emotions of shock, blame, denial, and depression, leaving parents overwhelmed by the magnitude of the situation:

*“I went into… severe depression… I used to get panic attacks (Liza).”*

*“I was thinking to hurt myself (John).”*

Parents felt relieved as they understood their child’s behavior and could move forward. Liza explained:

*“I did feel relief. I understand. I know he is not a naughty child. The realization that parents were not to blame was liberating.”*

#### 3.1.2. Parent’s Anxiety and Body Exhaustion

Mothers reported that they felt nervous, and had bodily exhaustion. Anna commented that:

*“He takes all my strength, efforts and make me easily nervous. I swear to God I do everything to accommodate his needs and ignore challenging behaviors but I am so tired to continue doing so.”*

Mothers are overwhelmed with the many responsibilities; Liza commented:

*“I am so stressed having three children to look after them. I have no time to relax and think.”*

Parents’ relationships with their neurotypical children were compromised, as they were mostly involved with the autistic child. Mothers expressed guilt about spending less time with their neurotypical children, but explained that neurotypical children understood their commitments:

*“I cannot remember him at seven… eight… nine, I remember the sadness in his face, but I cannot remember anything [else]” (Liza). One neurotypical child reported to his parents that he felt “left out and unloved” as Nick explained.*

In addition, the demands of a young child with ASD affected spousal relationships:

*“Initially, we were blaming each other later, I was blaming myself. The family becomes dysfunctional; you blame your husband” (Hellen). A change to the couple relationship was unavoidable due to the investment of time with the child. There is no time together as a couple.”*

Difficulties relating to self-care activities were distressing, especially as parents were aware of their mortality:

*“When he comes out of the bathroom… he does not care who is there. He will just run… now he is bigger (Helen). How long we will live and who will take care of her?” (Liza).*

Sensory overstimulation resulted in meltdowns, which reinforced decisions to stay home:

*“I cannot let him go to parties… if he gets a meltdown… people will not understand” (John).*

Difficulties with communication were another stressor, as parents did not know what was wrong:

*“It is not easy. Sometimes you just do not know… if he is hungry, if he needs to go to the toilet” (Nick).*

Changes to routine generated stress for the checklist for autism spectrum disorder (CASD):

*“She goes to school with the school transport; if the transport does not come, she would not want to go” (Nick).*

Families had to ensure that routines were in place but sometimes had no control over the situation.

#### 3.1.3. Using Distance Learning at Home: A Better Understanding of Children’s Abilities

Parents valued the benefit of being close to their children during online sessions at home. They got to know their children’s strengths and weaknesses. To Liza, online learning was:

*“A blessing gift for her child to proceed to learn and be engaged with activities during the lockdown period at home.”*

In addition, some of them were surprised by the abilities they had and never knew about before. Asma commented:

*“He surprised me. My husband came to the room saw him interacting with the teacher on the screen and communicating on activities and he asked me surprisingly: do you know that he could do that?”*

However, not all experiences were good. Helen indicated that her child’s behavior could not be controlled at home. He could not sit for more than 10 min in front of the computer or a desk to do his homework. She commented:

*“He moves all the time and she cannot afford him to sit and listen all the time, … I invent my strategy to let him sit…* *. I sit and put him on my lap and then hug him with my legs and hands so that he stays in place and concentrates on what he is learning… but I get tired I did not pursue… It is difficult for the Autistic child with ADHD child to sit at all scheduled times; he gets bored easily even crying and gains nothing.”*

Helen commented:

*“I feel a lot of stress, body pain, and confused brain. I always feel guilty. If I worked with my son, I did not check on his other brothers or I did not check with the baby. I blame myself every day and get angry if I missed one of his assignments not finished. I feel I am running every single day from 5 a.m. to 8 p.m. I do not stop running… Running…. no break until I fall asleep of tiredness.”*

It is worthy to note how the disability itself affects the whole family; it casts shadows of the usual stresses of accomplishing the learning process. The impulsivity of children adds another burden to the parents’ worry and anxiety. Children with ADHD are usually prone to accidents, as mentioned by a mother’s comment that her child fell from moving her brothers’ Jeb car while they were on a picnic, running after his falling slippers. In addition to this, the challenge becomes worse when it is accompanied by another disability, such as ADHD or sensory problems. This was noticed from the replies of Helen, Liza, and Asma. Although with Asma the difficulties were not so obvious, this may have been because of her good economic status and level of education.

Some of the difficulties mentioned during the interviews were related to the child’s disability, whereas others were related to school and the learning process, and others were related to the parents themselves (Lassoued et al. 2020). Children with ASD have unique characteristics that should be considered while learning online. These children should be taught according to their characteristics, having a continuous break between classes. Another suggestion by parents was that awareness workshops should be given to the societal community about Autism and the importance of taking into consideration the characteristics of included students. Financial aids and online learning resources such as computers should be devoted to parents who cannot afford them, and technical guidance should be provided to parents to improve their practical skills in supporting their children at home.

Parents’ perceived anxiety was one of the findings of the study related to children’s characteristics, as the severity of the behavioral problems affected the stress levels in parents. Thus, the whole family should be supported psychologically and socially to complete their role effectively and enjoy life. In addition, mothers with more than one child and greater responsibilities are more in need of this support.

## 4. Discussion

The study aimed to identify possible relationships between ASD children’s severity of symptoms and several other factors known to play a significant role in the levels of stress among parents with children with developmental disabilities.

As Miranda et al. (2019) point out, although, during the last decade, an increase in the prevalence of parental stress in this population has been reported (Baio et al. 2018), nonetheless, scarce research exists which thoroughly explores the relationship between risk and protective factors and parental stress. According to Kiami and Goodgold (2017), parental stress of children with ASD has been found to reach clinically significant levels in 77% of the cases, and is greater than the stress of parents of children with typical development (Giovagnoli et al. 2015; Rao and Beidel 2009; Davis and Carter 2008). Moreover, it largely exceeds the parental stress of children with other neurodevelopmental disorders (e.g., ADHD, specific learning disorders, intellectual disabilities, etc.) (Gupta 2007; Hayes and Watson 2013; Watson et al. 2013; Craig et al. 2016; Barroso et al. 2018). In general, the findings of the current study were consistent with those reported among the relevant literature (Feldman and Werner 2002; Gray 2003; Hutton and Caron 2005; Mancil et al. 2009).

According to the overall results, the significant correlation values detected among most variables were largely expected. Data analysis indicated that the level of the child’s functioning and his/her behavioral difficulties were significantly correlated with parental stress. There were significant correlations between scores on self-regulation problems reported from the teachers and the total parental stress scores and the mean scores on the subscale difficult child reported by the parent, a research finding that confirmed both of our hypotheses.

Additionally, most of the findings coincided with the majority of previous relevant literature. More specifically, the significant association found between higher levels of parental stress and the increase in the core symptomatology of ASD was in line with other studies, which emphasized a strong correlation between the severity of autism symptoms and higher levels of parental stress (Miranda et al. 2019; Ben-Sasson et al. 2013; Tomeny 2017; Bitsika and Sharpley 2017, etc.). The same finding validated the fact that positive and problem-focused strategies strongly correlate with less severe symptoms of ASD, which is also in line with the findings of Kiami and Goodgold (2017), Lai et al. (2015), Obeid and Daou (2014), and Benson (2010).

The literature revealed that each developmental disability, due to its unique behavioral characteristics, was itself a source of continuous anxiety for parents, as was the parent’s perception of the actual disability (Baio et al. 2018; Barroso et al. 2018; Stanojevic et al. 2017; Benson 2010) etc. Especially regarding ASD, problematic behaviors may include physical aggression, self-injury, property destruction, stereotyped behaviors, tantrums, etc. As a result, children with ASD are often highly disruptive to the classroom, home environments and the community (Horner et al. 2002; Efstratopoulou et al. 2012a). All of these behaviors have been directly related to parental stress (Kiami and Goodgold 2017; Barroso et al. 2018; Mancil et al. 2009). Zaidman-Zait et al. (2017), stated that mothers experienced lower levels of stress when they utilized more active coping strategies and relied less on disengaged coping strategies, either at the time of diagnosis or overtime.

On the other hand, parental stress was negatively correlated with the level of social functional support reported by the parents. This finding validates previous studies arguing that social support can significantly reduce the anxiety and distress experienced by parents raising a child with ASD (Lindsey and Barry 2018). In particular, Boyd (2002) found that a common coping strategy that decreased parental stress in this subgroup was contact with family members and parents of other children with autism. However, in the cases in which autism symptoms were more intense, it was found that parents were more reluctant to share their intimate feelings with other people. As a result, in these cases, the problem-focused coping strategies tended to reduce their effectiveness. These parents also declared that they received less empathy and caring from social sources.

Previous literature investigating the coping strategies used by parents of children with ASD to deal with various daily stressors has revealed conflicting findings, especially with regard to the long-term effectiveness of specific strategies often addressed by parents (e.g., social withdrawal, separating the child with ASD from his/her siblings, etc.). These findings can serve as useful guidelines to researchers and offer practical advice for intervention planning for practitioners working with families dealing with ASD, who often exhibit increased levels of stress (Boyd 2002).

Furthermore, correlation analyses also revealed that parental stress was positively associated with children’s high scores on the MBC subscale (rated by their teachers), which confirmed the high prevalence and intensity of ASD symptoms. This finding is in agreement with other studies that have reported strong correlations among ASD children’s problematic behaviors (rated with the use of several behavioral screening scales by parents and/or teachers), and the severity of ASD symptoms and parental stress (Posserud et al. 2018; Helland and Helland 2017). The strong link between parental stress and the emotional/behavioral disorder (EBD) of children with ASD found in this study has also been highlighted in other studies (Yorke et al. 2018; Barroso et al. 2018). This positive association of stress seems to exceed the value for the relationship between ASD symptom severity and EBD, which was also evident in the correlation analyses of this study (Miranda et al. 2019).

The findings from this study revealed a strong correlation between parental stress and children’s high scores on the MBC subscale. The higher the score on the MBC, the higher the level of parental stress. In addition, this correlation was also beneficial in terms of the issue of cross-informant agreement (i.e., the agreement between different informants’ multiple sources of information, e.g., parents, teachers, children or youth themselves) about a child’s overall functioning in different settings for the MBC (Achenbach et al. 2017). Little research is available in the area of cross-informant agreement (especially among parents and teachers). More specifically, even though, in this study, children were rated with the MBC only by their teachers, nevertheless, in this special online learning situation (due to the lockdown), parents and teachers had the unique opportunity to simultaneously observe children’s behaviors in exactly the same context.

As a result of the cross-informant agreement mentioned above, a unique common view of teachers and parents of children with ASD could be used as a valuable source for a better understanding of how to intervene to alter problematic behaviors of ASD children in both school and home settings. This will provide parents with better, more effective coping skills to deal with these behaviors. Likewise, the positive relationship between ASD symptoms and behavioral problems was confirmed, as consistently reported in the literature (Helland and Helland 2017; Posserud et al. 2018).

The ASD symptoms, as expected, were significantly and negatively associated with engagement coping and with social support suggesting that mothers who perceive the autism symptoms of their children with greater intensity tend to reduce their problem-focused coping strategies think they can communicate their intimate feelings to other people less, and receive fewer demonstrations of caring and empathy. Regarding parenting stress, the correlations generally support our hypotheses about the expected relationships. In addition to correlating with ASD symptoms, parenting stress presented a positive association with behavioral problems, exceeding the value for the relationship between ASD symptoms and behavioral problems.

Parents must receive help through family-centered supportive services that offer counseling, to decrease their stress levels by using appropriate coping strategies and other resources. Brief interventions that include stress management, details about specific behavioral impairments, and principles of behavior management within a set of components (information on autism, strategies for teaching new skills, improving social interaction and communication, service availability, family and community responses to autism) have shown their effectiveness in reducing parenting stress and improving family life (Kasari et al. 2015). Furthermore, according to an emerging body of research, mindfulness-based interventions may help reduce parenting stress in mothers who have children with ASD (Conner and White 2014).

### Limitations and Future Research

Even though the present study provided some innovative conceptualizations concerning the parental stress of children with ASD and the role of ASD symptom severity, self-regulation and coping strategies on parental stress, the study has some limitations. Addressing those limitations may lead to further research in this field. First, since the sample was relatively small, future studies could include larger samples to generalize findings.

In addition, future research should include cross informant ratings with the MBC (and other similar screening tools) of both parents and teachers of children (and adolescents) with ASD and other developmental disorders during distance learning situations. This would further validate the cross-informant agreement of such instruments and lead to better intervention strategies and techniques for ASD children’s learning, which will derive from the common experiences of parents and teachers.

Apart from parental educational levels, other factors could also be included in future research, such as socioeconomic status, everyday life conditions, etc. Finally, findings from similar studies can have many practical implications, in terms of awareness planning, special education training workshops and communication skills training for parents of children with ASD and other developmental difficulties. This could reduce the anxiety levels reported by parents of ASD and other developmental disorders and make them feel more confident to support their children.

## 5. Conclusions

As the literature suggests, the issue of parental stress and their psychological adaptation in the extremely difficult situation of rearing a child with ASD is a very complex variable, which depends upon a combination of both risk and protective factors. These include the personal characteristics and behavioral profile of the child, the severity of the core symptomatology of ASD, the frequency and severity of emotional and behavioral difficulties manifested, the family’s positive and negative coping strategies, as well as the level of social and/or other types of support (e.g., educational) parents receive, especially in stressful situations.

In line with previous research, the present study also revealed that both ASD symptomatology and EBD were highly correlated with high levels of anxiety in parents, whereas engagement coping, sufficient or high educational level and social functional support were factors, which negatively correlated with parental stress. Likewise, findings confirmed that the prevalence of less severe ASD symptoms and better self-regulation skills for the children werepositively correlated with coping strategies used by parents and, consequently, with a reduced anxiety level. In addition, results confirmed the mediating role of EBD, parents’ coping strategies and social functional support in the association between parental stress and the symptom severity of children with ASD, also reported in previous studies.

Hence, eliminating the stressors parents face in raising children with ASD does not seem possible. Instead, improving parental coping and resilience should be the objective in helping family functioning when there are new and ongoing challenges.

Finally, the present study points out the need to promote parents’ coping orientation and the application of behavioral strategies with their children to help them handle the immense impact of stress. Suggestions to support families with children with ASD aim at the development of strategies for the improvement of the self-regulation skills of nonverbal children with ASD. This may be particularly important in terms of reducing parental stress for families of children with autism and other developmental disabilities.

## Figures and Tables

**Table 1:** Parents’ Demographic characteristics.

	N	%
**Gender**	75	
Male		27%
Female		83%
**Marital status**		
Married		80%
Divorced		14%
Single—No Family		6%
**Educational Level**		
University Level		53.3%
Secondary Level		40%
Primary/No Education		6.7%
**Working Status**		
Currently Working		67%
Unemployed or never worked in the past		33%

**Table 2:** Intercorrelations between parental stress and self-regulation problems in ASD Children (N = 75).

Variable	Mean	SD	Parental Stress	Parent–Child Dysfunction	Difficult Child	Defense Responses	Total Parental Stress	Self-Regulation Scores in Children
Parental stress	25.2	4.8	–	0.486	0.394	0.738 **	0.704 **	0.297
Parent–Child Dysfunction	26.0	5.8	0.486	–	0.592 *	0.392	0.651 **	0.292
Difficult child	25.0	5.8	0.394	0.592 *	–	0.623 *	0.618 **	0.761 **
Defense Responses	17.3	3.3	0.738 **	0.392	0.623 *	–	0.675 **	0.661 *
Total Parental Stress	87.0	17.2	0.704 **	0.651*	0.618 **	0.675 **	–	0.713 **
Self-regulation scores in children	29.7	5.7	0.297	0.292	0.661 *	0.349 **	0.713 **	–

Note: ASD = autism spectrum disorders, ** Correlation is significant at the 0.01 level (2-tailed), * Correlation is significant at the 0.05 level (2-tailed).

## Data Availability

The data presented in this study are available on request from the corresponding author due to privacy issues. The data are not publicly available due to privacy.

## Abbreviations

The following abbreviations are used in this manuscript:

ABA	Applied Behavior Analysis
ADHD	Attention Deficit Hyperactivity Disorder
ADI-R	Autistic Diagnostic Interview-Revised
ASD	Autism Spectrum Disorder
CASD	Checklist for Autism Spectrum Disorder
DSM-5	Diagnostic and Statistical Manual of Mental Disorder-5
EBD	Emotional/Behavioral Disorder
ICC	Intraclass Correlation Coefficients
IQ	Intellectual Quotient
LEAP	Learning Experiences: An Alternative Program for Preschoolers and Parents
MBC	Motor Behaviour Checklist for children
PECS	Picture Exchange Communication System
PSI-SF	Parenting Stress Index–Short Form
TEACCH	Treatment and Education of Autistic and related Communication handicapped Children
SDQ	Strengths and Difficulties Questionnaire
SPSS	Statistical Package for Social Science
